# High level of intraoperative lactate might predict acute kidney injury in aortic arch surgery via minimally invasive approach in patients with type A dissection

**DOI:** 10.3389/fcvm.2023.1188393

**Published:** 2023-07-04

**Authors:** Ying Lyu, Yu Liu, Xiong Xiao, Zhonglu Yang, Yuguang Ge, Hui Jiang

**Affiliations:** ^1^ Graduate School, China Medical University, Shenyang, China; ^2^ Department of Cardiovascular Surgery, General Hospital of Northern Theater Command, Shenyang, China

**Keywords:** aortic dissection, total arch replacement, acute kidney injury, minimally invasive approach, lactate

## Abstract

**Background:**

A high incidence of acute kidney injury (AKI) has been recorded in total arch replacement (TAR) combined with frozen elephant trunk (FET) implantation in patients with acute type A dissection (ATAAD) via median sternotomy approach with some risk factors. However, the independent risk factors for AKI via a minimally invasive approach have not yet been identified.

**Methods:**

A total of 207 patients with ATAAD were enrolled from January 2018 and November 2019 and were divided into AKI and non-AKI groups. The current surgical strategy was TAR combined with FET via a single upper hemisternotomy approach, a minimally invasive method. An increase in the serum creatinine (Cr) level to ≥2 times the baseline level 48 h post-surgery was defined as AKI. The morbidity of AKI was investigated with a step-by-step backward multivariate analysis of its independent risk factors and a receiver-operating characteristic curve analysis.

**Results:**

Postoperative AKI was observed in 39 (18.8%) patients, and the total hospital mortality was 8.7%. Univariate analysis found that preoperative Cr, weight, circulatory arrest time ≥60 min, intraoperative highest lactate (Lac), and intraoperative transfusion had significant differences between the two groups. However, multivariate step-by-step backward logistic regression analysis identified intraoperative highest Lac and transfusion as independent risk factors for postoperative AKI and intraoperative highest Lac was identified as the most critical independent risk factor estimated by the partial chi-square statistic minus the predicted degrees of freedom with 4.3 mmol/L as the optimal cut-off point for prediction for AKI.

**Conclusions:**

Intraoperative highest Lac and transfusion were independent risk factors for postoperative AKI, which led to high hospital mortality. Moreover, intraoperative highest Lac was the most critical independent risk factor and high level of intraoperative highest Lac (4.3 mmol/L) might predict for postoperative AKI.

## Introduction

Total arch replacement (TAR) combined with frozen elephant trunk (FET) implantation has become a routine procedure for acute type A aortic dissection (ATAAD) in China (1, 2), which also has been carried out via a single upper hemisternotomy approach (UHS) as a minimally invasive practice (3). Based on the limited reports on the minimally invasive procedure of ATAAD (4–6), TAR combined with FET was considered safe with high efficacy and similar mortality and morbidity.

Acute kidney injury (AKI) is a common postoperative complication after TAR, with morbidity ranging from 36% to 66.7% (7). Postoperative AKI has been demonstrated as an independent risk factor for mortality after TAR combined with FET, which also has an adverse effect on long-term postoperative survival (8, 9). Several studies based on median sternotomy approach to operative AKI have revealed that circulatory arrest (CA) time, lactic acidosis, blood transfusion, high body mass index (BMI), advanced age, and perioperative sepsis might be the independent risk factors for postoperative AKI (7, 10–12). However, only a few studies have focused on TAR with FET via a minimally invasive incision with no study on the risk factors for AKI.

In the present study, a retrospective analysis was carried out to search for the risk factors for postoperative AKI after minimally invasive TAR, which might be beneficial for improving the clinical effects of the procedure, especially in lowering the morbidity of AKI.

## Methods

This study was approved by the Ethics Committee of the General Hospital of Northern Theater Command, Shenyang, China (Y2022.210). It was conducted according to the ethical guidelines published by the Helsinki Declaration. All the patients provided informed consent.

### Patients’ data

We retrospectively reviewed our surgical outcome for 213 consecutive patients with ATAAD who underwent TAR combined with FET implantation via a single upper hemisternotomy approach at the General Hospital of Northern Theater Command, Shenyang, China, between January 2018 and November 2019. Subsequently, the patients were divided into AKI and non-AKI groups. The same surgeons, anesthetists, perfusionists, and cardiologists completed the surgeries and this study. All patients were diagnosed definitively as acute type A aortic dissection based on computed tomography angiography (CTA). The exclusion criteria were as follows: stroke, malperfusion syndrome, and some concomitant operations that needed full sternotomy, and subemergency surgeries were tended to be carried out unless complications suffered were life-threatening.

### Surgical procedure

The procedure of TAR combined with FET was performed via a single UHS as described previously (3, 6, 13). Briefly, the UHS was made from the sternal notch to the level of the fourth intercostal space and then extended to the right fourth intercostal space. Cardiopulmonary bypass (CPB) was started with innominate artery (IA) cannulation and right atrium cannulation with left ventricular venting with 2.0–2.4 L/min/m^2^ of systemic perfusion flow. Myocardial protection was carried out by antegrade cardioplegia through the aortic root or coronary orifices after aortotomy with cold blood cardioplegia. The bilateral selective antegrade cerebral perfusion was performed by IA and left common carotid artery (LCA) after IA was cross-clamped with 5–10 ml/kg/min of cerebral perfusion flow. When aortic root procedures were carried out, and the nasopharyngeal temperature reached 28°C, moderate hypothermia circulatory arrest (MHCA) was effectuated for FET and TAR with a stent graft (MicroPort Medical Co. Ltd., Shanghai, China), and a four-branch prosthetic graft (VASCUTEK Ltd., a Terumo Company, Inchinnan, Scotland, United Kingdom). After arch arteries were transected and the proximal segment was sutured, FET was inserted into the true lumen of the distal aorta, which was firmly attached to the distal end of the four-branch prosthetic graft. After the anastomosis was completed, lower body perfusion (LBP) was restarted via the perfusion limb of the four-branch prosthetic graft. Then, LCA, proximal aortic stump, left subclavian artery, and IA were anastomosed to the prosthetic graft successively, rewarming was started, and CPB was terminated gradually.

### Data collection

Clinical records and data on patient demographics, details of medical and surgical treatments, and patients’ outcomes were collected for the study. AKI was defined as an increase in creatinine (Cr) level to ≥2 times the baseline level in 48 h post-surgery (14), which is equivalent to the thresholds of “injury” in the RIFLE criteria by the Acute Dialysis Quality Initiative group, “stage 2” based on the Acute Kidney Injury Network (AKIN) and “stage 2” in the Kidney Disease Improving Global Outcomes (KDIGO) guideline. Intraoperative blood transfusion was defined as intraoperative transfusion of red blood cells. The criteria of transfusion were hematocrit <0.21 during CPB and <0.3 after CPB (15).

### Statistical analysis

All the analyses were performed using SPSS version 22.0 software (SPSS Inc., Chicago, IL, United States). Logistic regression analyses were used to identify the independent risk factors of AKI. The normally distributed data are presented as group mean ± SD, and non-normally distributed data are presented as the median and interquartile ranges. AKI and non-AKI groups were evaluated using unpaired two-tailed Student's *t*-test (normally distributed data) or Mann–Whitney *U*-test (non-normally distributed data) for the continuous variables. Categorical variables were analyzed by *χ*^2^ test or Fisher's exact probability test (if necessary). *P* < 0.05 indicated a statistically significant difference. Variables with significant differences (*P* < 0.05) were analyzed by univariate analysis, and only those with statistical significance were used for step-by-step backward multivariate logistic regression analyses. A receiver-operating characteristic (ROC) curve was applied to determine the optimal cut-off value of viabilities in the prediction of AKI. The importance of each screened risk factor in the full model was estimated by the partial chi-square statistic minus the predicted degrees of freedom (*χ*^2^ − df). Features selection and final fitting were evaluated depending on Akaike information criterion (AIC) values.

## Results

A total of 207/213 patients were enrolled in this study, and 6 patients with neurological complications (2 cases), malperfusion syndrome (1 case), and concomitant operations (3 cases) were excluded. The perioperative characteristics of 207 patients are shown in Table 1. Of the 207 patients, 39 (18.8%) suffered from AKI, and 18 (8.7%) hospital deaths were caused by postoperative stroke (7 cases), postoperative multiple organ failure (4 cases), sudden hemodynamic changes (4 cases), and malignant arrhythmia (3 cases).

**Table 1:** Perioperative variables of patients.

Variables	AKI group (*n* = 39)	Non-AKI group (*n* = 168)	*P*-value
Preoperation			
Male	32 (82.1%)	120 (71.4%)	0.176
Age (years)	49.4 ± 11.4	50.5 ± 11.0	0.757
Weight (kg)	82 (71, 95)	75 (67, 85)	0.018
Hypertension	32 (82.1%)	128 (76.2%)	0.431
Hyperlipidemia	1 (2.6%)	8 (4.8%)	0.544
Diabetes	3 (7.7%)	6 (3.6%)	0.256
Smoking history	24 (61.5%)	82 (48.8%)	0.152
Preoperative Cr (μmol/L)	81.90 (65.00, 121.00)	70.50 (58.25, 87.50)	0.012
Preoperative Lac (mmol/L)	1.5 (1.2, 2.0)	1.5 (1.2, 1.9)	0.348
LVEF (%)	58.00 (57.00, 58.00)	58.00 (57.00, 59.75)	0.630
Intraoperation			
Aortic valvuloplasty	13 (33.3%)	48 (28.6%)	0.557
Aortic valve replacement	0 (0.0)	6 (3.6%)	0.231
Bentall	7 (17.9%)	27 (16.1%)	0.776
CPB time (min)	177 (153.00, 200.00)	163.00 (147.25, 190.00)	0.182
Cross-clamp time (min)	107.00 (85.00, 115.00)	94.00 (80.00,111.75)	0.280
CA time (min)	32.00 (30.00, 43.00)	32.00 (25.00, 38.00)	0.216
Min nasopharyngeal T (°C)	26.00 (24.00, 27.70)	26.65 (25.00, 27.88)	0.116
CA time ≥60	3 (7.7)	2 (1.2)	0.017
Intraoperative transfusion	24 (61.5%)	66 (39.3%)	0.012
Intraoperative highest Lac (mmol/L)	4.90 (4.50, 5.40)	4.50 (3.90, 5.10)	0.004
Intraoperative urine volume (ml)	650 (250, 1,170)	750 (405, 1,200)	0.198
Intraoperative blood loss (ml)	500 (350, 600)	500 (350, 600)	0.375
Postoperation			
Postoperative low cardiac output	4 (10.3%)	10 (6.0%)	0.335
Reoperation for bleeding	0 (0.0)	4 (2.4%)	0.331
Reventilation	2 (5.1%)	4 (2.4%)	0.357
Stroke	2 (5.1%)	5 (3.0%)	0.503
Drainage volume (mL)	780 (540, 1,220)	777 (496, 972)	0.208
Ventilation time (h)	40 (19, 68)	20 (17, 42)	0.019
ICU stay time (h)	83 (42, 133)	42 (28,67)	0.001
Postoperative hospital stays (day)	17 (13, 21)	15 (11, 20)	0.139
Mortality	7 (17.9%)	11 (6.5%)	0.023

LVEF, left ventricular ejection fraction; Min nasopharyngeal T, minimum nasopharyngeal temperature; ICU, intensive care unit.

Patients were divided into AKI (*n *= 39) and non-AKI groups (*n* = 168). As shown in Table 1, significant differences were observed between AKI and non-AKI groups in preoperative Cr [81.9 (65.0–121.0) µmol/L vs. 70.5 (58.3–87.5) µmol/L, *P* = 0.012], weight [82 (71–95) kg vs. 75 (67–85) kg, *P* = 0.018], circulatory arrest time [≥60 min (7.7% vs. 1.2%, *P* = 0.017)], intraoperative highest lactate (Lac) [4.9 (4.5–5.4) mmol/L vs. 4.5 (3.9–5.1) mmol/L, *P* = 0.004], intraoperative transfusion (61.6.% vs. 39.3%, *P* = 0.012), ventilation time [40 (19–68) h vs. 20 (17–42) h, *P* = 0.019], length of intensive care unit (ICU) stay [83 (42–133) h vs. 42 (29–67) h, *P* = 0.001], and hospital mortality (17.9% vs. 6.5%, *P* = 0.023).

As shown in Table 2, multivariate analysis of postoperative AKI identified by logistic regression analysis showed that independent risk factors were intraoperative highest Lac [odds ratio (OR): 1.345; 95% confidence interval (CI): 1.072–1.687] and intraoperative transfusion (OR: 2.220; 95% CI: 1.049–4.698). After the partial chi-square statistic minus the predicted degrees of freedom (5.579 on intraoperative highest Lac vs. 3.343 on intraoperative transfusion), the intraoperative highest Lac was found as the most critical risk factor for AKI. The AIC of the final model that kept lactate and intraoperative transfusion as predictors was 97.158.

**Table 2:** Independent risk factors for postoperative AKI.

Variables	Univariate *P*-value	Multivariate			*χ*^2^ − df
		*P*-value	OR	95% CI	
Preoperative Cr (µmol/L)	0.016	0.073	1.007	0.999–1.014	
Weight (kg)	0.024	0.060	1.024	0.999–1.050	
CA time ≥60 (%)	0.038	0.943	1.087	0.110–10.717	
Intraoperative highest Lac (mmol/L)	0.008	0.010	1.345	1.072–1.687	5.579
Intraoperative transfusion (%)	0.013	0.043	2.220	1.049–4.698	3.343

**Figure 1:**
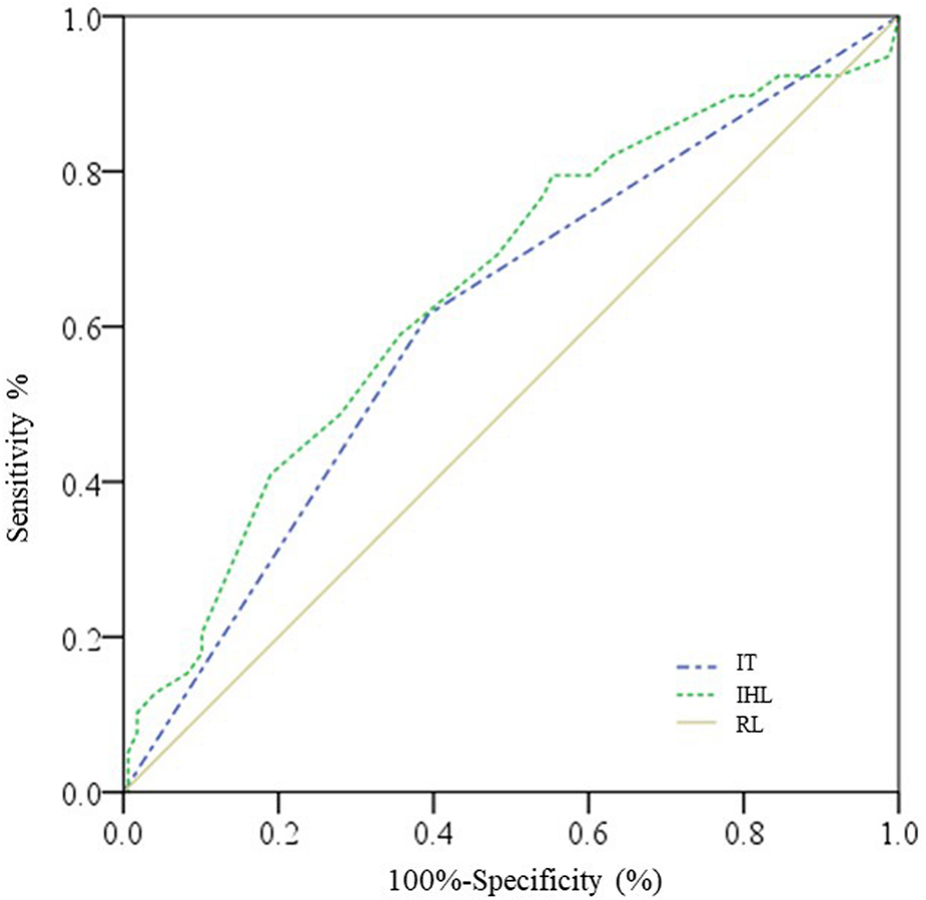
ROC curves for intraoperative highest Lac and intraoperative transfusion. IHL, intraoperative highest lactate; IT, intraoperative transfusion; RL, reference line.

The cut-off point of intraoperative highest Lac was 4.3 mmol/L with a sensitivity of 79.5% and a specificity of 44.6% for the prediction of AKI, while the area under the ROC curve was 0.648 (95% CI: 0.549–0.746, *P *= 0.004) (Figure 1).

## Discussion

The main findings of this study were that intraoperative highest Lac level was the most critical independent risk factor for AKI after surgery for ATAAD via a minimally invasive approach. Furthermore, ventilation time, length of ICU stays, and hospital mortality were significantly higher in the AKI group than in the non-AKI group, indicating that postoperative AKI leads to poor clinical outcomes, as reported previously (7, 16).

Postoperative AKI is a common complication after surgery for ATAAD based on studies via the median sternotomy approach, which might cause poor clinical outcomes and have some perioperative independent risk factors (7, 9, 11, 12, 14). However, studies above mentioned were all based on median sternotomy approach and no study was concerned about the risk factor of AKI on minimal invasive incision. In the present study, the risk factor of AKI on a single upper hemisternotomy approach was carried out and the total morbidity of postoperative AKI was 18.8%, which was lower than that in a previous study (7) and might be attributed to different definitions of AKI and influence by minimal invasive incision. Amano et al. (14) demonstrated that age, BMI, chronic kidney disease in preoperation, and lower body circulatory arrest time during the operation were independent predictors for postoperative AKI. Moreover, Nota et al. (12) revealed that the circulatory arrest time >60 min was an independent risk factor for postoperative AKI. Hence, we selected the morbidity of circulatory arrest time ≥60 min as a critical variable in our study. The results showed higher preoperative Cr [81.9 (65.0–121.0) μmol/L vs. 70.5 (58.3–87.5) μmol/L, *P* = 0.012] and weight [82 (71–95) kg vs. 75 (67–85) kg, *P* = 0.018] in preoperative characteristics, whereas intraoperative highest Lac level [4.9 (4.5–5.4) mmol/L vs. 4.5 (3.9–5.1) mmol/L, *P* = 0.004], higher incidence of circulatory arrest time ≥60 min (7.7% vs. 1.2%, *P* = 0.017), and intraoperative transfusion (61.6.% vs. 39.3%, *P* = 0.012) were risk factors identified by univariate analysis. Some results were consistent with previous studies described above, including intraoperative highest Lac level and perioperative transfusion (10, 11). These two studies reported that transfusions of packed red blood cells, platelet counts, and lactic acidosis increased the incidence of AKI, respectively. However, in multivariate analysis, only intraoperative highest Lac (OR 1.345; 95% CI: 1.072–1.687) and intraoperative transfusion (OR: 2.220; 95% CI: 1.049–4.698) were demonstrated as independent risk factors for postoperative AKI. Based on the partial chi-square statistic minus the predicted degrees of freedom, the intraoperative highest Lac was the most critical risk factor. Together, these results suggested that the independent risk factors for postoperative AKI were similar irrespective of the minimally invasive approach and median sternotomy approach. Moreover, ROC analysis of intraoperative highest Lac was carried out and 4.3 mmol/L of intraoperative Lac was found as an optimal cut-off point with a sensitivity of 79.5% and a specificity of 44.6% for the prediction of AKI. Therefore, maintaining Lac below 4.3 mmol/L intraoperation might diminish the incidence of postoperative AKI.

Blood loss is common in TAR surgery, frequently requiring blood transfusion, which has been shown to be associated with AKI in patients undergoing CPB (16). However, transfusion rates vary greatly between different institutions, with only a few studies in patients with ATAAD undergoing TAR (10, 17). In the current study, the total intraoperative transfusion rate was 46.4% with 69.2% in the AKI group and 41.1% in the non-AKI group, which was consistent with the study reported by Li et al. (10). This phenomenon could be reason why transfusion with adverse outcomes might involve impaired oxygen delivery, decreased deformability of stored red blood cells, prothrombotic effects from the increased release of procoagulant factors, and transfusion-related immunosuppression (18, 19). Hyperlactatemia is a predictor of outcomes after ATAAD surgery (20), which reflects prolonged tissue hypoperfusion or increased oxygen utilization and might lead to postoperative AKI (21, 22). In TAR surgery, circulatory arrest caused by tissue hypoperfusion might be related to hyperlactatemia. In the present study, intraoperative highest Lac level was found to be an independent risk factor for postoperative AKI. However, no significant difference between the two groups was detected in the circulatory arrest time, which might be caused by a short circulatory arrest time and a small sample size.

The problem of intraoperative transfusion and intraoperative highest Lac level could be resolved as follows. For perioperative transfusion-related AKI, one strategy is avoiding unnecessary blood transfusion and prioritizing reducing the rate of transfusion. Another strategy is to improve the quality of the transfused blood. Washing the blood might be a beneficial method to remove the proinflammatory molecules, free hemoglobin, and iron that accumulates in the supernatant during storage (23). Finally, choosing an effective and simple surgical procedure might be beneficial for high-risk patients (10). For the intraoperative highest Lac level-related AKI, the main solution might reduce the circulatory arrest time, which can be resolved by LBP through various ways (6, 24, 25). Since the circulatory arrest time indicated hypoperfusion to the lower body, which raises the Lac level, shortened circulatory arrest time might decrease the intraoperative highest Lac level.

### Limitation

Nevertheless, the present study has several limitations. First, the retrospective study design is a time-based comparison with a time bias. Second, we defined an increase in the Cr level to ≥2 times the baseline level as AKI, as referred to in a previous study (14). Therefore, the AKI diagnosed in our study was severe and might show reduced morbidity of AKI. Third, this is a monocenter study with a small sample size, and most patients were from Northeast China, which might cause a confounder bias. Finally, the long-term outcomes were not collected, and follow-up of all patients should be underway.

## Conclusion

Although TAR combined with FET via a single UHS showed favorable results, the morbidity of postoperative AKI is still high, which causes higher hospital mortality. Intraoperative highest Lac and transfusion were independent risk factors for postoperative AKI, which led to high hospital mortality. Moreover, intraoperative highest Lac was the most critical independent risk factor and high level of intraoperative highest Lac (4.3 mmol/L) might predict for postoperative AKI.

## Data Availability

The raw data supporting the conclusion of this article will be made available by the authors, without undue reservation.
